# Cross talk of tumor protein D52 (TPD52) with KLF9, PKCε, and MicroRNA 223 in ovarian cancer

**DOI:** 10.1186/s13048-023-01292-1

**Published:** 2023-10-13

**Authors:** Khushbukhat Khan, Sameen Zafar, Yasmin Badshah, Naeem Mahmood Ashraf, Mehak Rafiq, Lubna Danish, Maria Shabbir, Janeen H. Trembley, Tayyaba Afsar, Ali Almajwal, Suhail Razak

**Affiliations:** 1 Atta-ur-Rahman School of Applied Biosciences (ASAB), National University of Sciences and Technology (NUST), Islamabad, 44000 Pakistan; 2 School of Biochemistry & Biotechnology, University of the Punjab, Lahore, Pakistan; 3 School of Interdisciplinary Engineering & Sciences (SINES), National University of Sciences and Technology, Islamabad, 44000 Pakistan; 4 Agricultural Research Institute, Tarnab, Peshawar Pakistan; 5 Department of Healthcare Biotechnology, Atta-ur-Rahman School of Applied Biosciences, National University of Sciences and Technology, Islamabad, Pakistan; 6 Research Service, Minneapolis VA Health Care System, Minneapolis, MN USA; 7 Department of Laboratory Medicine and Pathology, University of Minnesota, Minneapolis, MN USA; 8 Masonic Cancer Center, University of Minnesota, Minneapolis, MN USA; 9 Department of Community Health Sciences, College of Applied Medical Sciences, King Saud University, Riyadh, Saudi Arabia

**Keywords:** Ovarian cancer, TPD52, Protein kinase C epsilon, microRNA

## Abstract

**Background:**

Gynecologic cancers comprise malignancies in the female reproductive organs. Ovarian cancer ranks sixth in terms of incidence rates while seventh in terms of mortality rates. The stage at which ovarian cancer is diagnosed mainly determines the survival outcomes of patients. Various screening approaches are presently employed for diagnosing ovarian cancer; however, these techniques have low accuracy and are non-specific, resulting in high mortality rates of patients due to this disease. Hence, it is crucial to identify improved screening and diagnostic markers to overcome this cancer. This study aimed to find new biomarkers to facilitate the prognosis and diagnosis of ovarian cancer.

**Methods:**

Bioinformatics approaches were used to predict the tertiary structure and cellular localization along with phylogenetic analysis of TPD52. Its molecular interactions were determined through KEGG analysis, and real-time PCR-based expression analysis was performed to assess its co-expression with another oncogenic cellular pathway (miR-223, KLF9, and PKCε) proteins in ovarian cancer.

**Results:**

Bioinformatics analysis depicted the cytoplasmic localization of TPD52 and the high conservation of its coiled-coil domains. Further study revealed that TPD52 mRNA and miRNA-223 expression was elevated, while the expression of KLF 9 and PKCε was reduced in the blood of ovarian cancer patients. Furthermore, TPD52 and miR-223 expression were upregulated in the early stages of cancer and non-metastatic cancers.

**Conclusion:**

TPD52, miR-223, PKCε, and KLF9, can be used as a blood based markers for disease prognosis, metastasis, and treatment response. The study outcomes hold great potential to be translated at the clinical level after further validation on larger cohorts.

## Background

Ovarian cancer (OC) is considered the deadliest gynecological disease in women. It develops in the epithelial cells of ovaries or fallopian tubes [1, 2]. OC is the seventh most prevalent type of cancer, with 150,000 reported deaths in the year 2020 globally [3–5]. Although survival rates among patients of OC are high if diagnosed at early stages because of advancements in treatments, the tumor is already metastasized before initial diagnosis in almost 70% of females [6–8]. Currently, there are no specific biomarkers for OC as the serum marker CA-125 is also elevated in various other conditions [9]. Hence, there is an urgent need to identify novel alternative approaches for screening OC.

The TPD52 gene is located on chromosome 8 at the region frequently amplified in various human cancers [10, 11]. Studies have reported the role of TPD52 in different cellular processes [12]. Increased expression levels of TPD52 are associated with multiple types of cancers, including breast, prostate, pancreas, and melanoma [13–15]. Studies have reported the role of TPD52 in different cancer signaling pathways, such as PI3K/Akt [16], PKB, and NFκB [17, 18], and p21 [19]. Similarly, studies have shown that miR-223 plays essential roles in regulating various genes and cancer progression [20–23]. Increased expression of miR-223 promotes abnormal activation of the Akt/mTOR pathway in several diseases, including pancreatic cancer and colorectal cancer [24].

KLF9 is a member of the KLF family of transcription factors and regulates cellular adhesion, differentiation, and proliferation processes in the endometrium [25–27]. Studies have shown that altered expression of KLF9 is involved in breast, prostate, and cervical cancer [28–30]. Moreover, KLF9 targets significant signaling pathways in various cancers [31–33]. PKCε is one of the members of the PKC family and is most widely studied for its contribution to malignant transformation [34]. PKCε is usually referred to as an oncogenic kinase as it is involved in the regulation of mitogenesis, cellular invasion, and survival. Over-expression of PKCε has been reported in various cancers [35–39]. Recent studies have revealed that the downregulation of PKCε is associated with the inhibition of Akt, resulting in a better prognosis in breast cancer [40].

The aim of this study is to predict the tertiary structure of TPD52 for the first time to provide a deep insight towards a better understanding regarding the functional structure as well as the molecular basis of TPD52 as well as other members of the TPD family. the 3-D tertiary structure of proteins assists in the understanding of their molecular functions as well as their interactions with their binding proteins. This study illustrates the approaches to determining the conserved domains and regions of TPD52 and the subsequent development of genetic pathways, thereby establishing a molecular cross-talk among TPD52 and its upstream as well as downstream elements. Additionally, we determined the co-expression of TPD52, KLF9, PKCε, and miRNA-223 and their relationship with the clinicopathological features of OC to explore the prognostic potential of these genes in patients with OC.

## Methods

### Blood sample collection

The ethical review board approved the current study of the parent institute ASAB, National University of Sciences and Technology. After oral as well as written informed consent at Combined Military Hospital, Rawalpindi, blood samples were obtained from OC patients (n = 150) that was confirmed by oncologists. Patients with HIV co-infection or any other co-morbidity such as cardiovascular disease, neurological or metabolic disorders were excluded. The median age of OC patients was 59 years (range 24–70 years). Blood samples from healthy individuals (n = 150) were taken for the control group (Table 1). The study was performed by following the protocols and principles of the Helsinki declaration on human subjects [41].

**Table 1:** Clinico-pathological features of cancerous patients enrolled in the study

Clinico pathological characteristics of patients		Ovarian cancer N (%)
Age	≤ 50	84 (56)
	> 50	66 (44)
Stage	I–II	54 (36)
	III–IV	96 (64)
Metastasis	Metastasis	60 (40)
	Non-metastatic	90 (60)
Treatment	Chemotherapy	150 (100)
	Radiotherapy	0
	Chemotherapy + Radiotherapy	0

### RNA extraction and cDNA synthesis

RNA was extracted from the whole blood of cancer samples using TriZol reagent (Thermo Fischer Scientific, Waltham, MA, USA) using the manufacturer’s instructions. The protocol was opted from the studies by Zahra et al. [42] and Khan et al. [43]. The reaction was conducted on ice to avoid RNA degradation. The RNA concentration was estimated from Nanodrop, and its purity was determined by an absorption ratio of 260/280. For cDNA synthesis, 20 µl of the reaction mixture was prepared by adding 1 µl of Oligo(dT), 1 µl dNTP MIX (2.5mM), < 5 µg of RNA, and RNAase-free water Up to 10 µl. The reaction mixture was incubated at 65 °C in a thermocycler for 5 min. In the next step, 10X reaction buffer (2 µl), 100mM DTT (1 µl), RNase inhibitor (0.5 µl), and RTase (1 µl) were added in a PCR tube (same) and put in a thermocycler for 50 min at 42 °C and 70 °C for 10 min. The synthesized cDNA was stored at − 20 °C.

### Real-time PCR

For the analysis of candidate genes and miR-223, RT PCR was employed. The conditions for real-time PCR opted from the study [42, 44] with little modifications. For the preparation of the RT PCR reaction mixture (total 20 µl), SYBR™ Green PCR Master Mix (Thermo Fisher Scientific Inc) 10ul, Sense, and Antisense primers (6 Mm) along with 10 µg cDNA were used. qPCR amplification conditions were: initial denaturation at 95 °C for 10 min, then 40 cycles of amplification where 95 °C denaturation for 15 s was followed by extension for 1 min at 61 °C, and real-time analysis for 45 s at 75 °C. Table 2 contains the information related to primer sequences. Primers’ specificity was determined by analyzing qPCR’s melt curve, and 7300 SDS software was used for analysis. Gene expression quantification was carried out using the 2-ΔΔCT method, where the cycle threshold (Ct) value was converted to fold-change using the Livak method. The experiment was performed in triplicates for results validation and β-actin was used as internal control.

**Table 2:** Sequences and parameters of primer used for qPCR

Name	Sequence	GC content %	Annealing Temperature
KLF 9 Forward	5′-TGGCTGTGGGAAAGTCTATGG-3′	52.4	60 °C
KLF 9 Reverse	5’-CTCGTCTGAGCGGGAGAACT-3	60	60 °C
TPD52 Forward	5-′GCTGCTTTTTCGTCTGTTGGCT-3′	50	60 °C
TPD52 Reverse	3′TCAAATGATTTAAAAGTTGGGGAGTT	30	60 °C
miR223 Forward	5′-AGCCGTGTCAGTTTGTCAAAT-3′	42.9	60 °C
miR-223Reverse	5′-GTGCAGGGTCCGAGG TC-3′	70.6	60 °C
PKCε Forward	5′-AGCCTCGTTCACGGTTCT-3′	55.6	60 °C
PKCε Reverse	5′-TGTCCAGCCATCATCTCG-3′	55.6	60 °C
*beta*-*actin Forward*	CACCATTGGCAATGAGCGGTTC	54.5	60 °C
*beta*-*actin Reverse*	AGGTCTTTGCGGATGTCCACGT	54.5	60 °C

### Statistical analysis

Ordinary one-way ANOVA performed statistical analysis to show the relationship between different clinicopathological features and expression of TPD52, KLF9, PKCε, and miR-223 in ovarian cancer. Analysis was performed using GraphPad prism 6.0 software. The significance was defined as p < 0.001. Moreover, GraphPad was also used to perform the receiver operating characteristic (ROC) curve analysis to determine the specificity of these biomarkers.

### Tertiary structure prediction

The tertiary structure of Tumor Protein D52 (TPD52) was determined through I-TASSER, which uses multiple threading approaches for automated prediction of protein 3-dimensional structure [45]. For this purpose, the amino acid sequence of TPD52 was retrieved from NCBI in FASTA format. To validate the predicted structure, Ramachandran analysis was performed through PROCKECK [46] to determine the right angles and confirmations of the constituent amino acid residues of the protein. Lastly, domains in the predicted TPD52 structure were identified through the InterPro database [47].

### Prediction of localization

The localization of TPD52 was predicted through DeepLoc 1.0. This tool predicts the sub-cellular trans localization of eukaryotic proteins. DeepLoc 1.0 is a multi-label prediction tool capable of predicting more than one cellular localization for a given protein. It can differentiate between up to ten different localization sites. Moreover, this tool also indicates other sorting signals that influence the sub-cellular localization of target protein [48].

### Phylogenetic analysis

TPD52 is a member of the TPD family proteins with sequence and structural similarities with the other proteins of its family. Therefore, to identify the conservation of amino acid sequence as well as the evolutionary relationships among all members of the TPD family protein, multiple sequence alignment (MSA) was performed using Clustal W [49], and a phylogenetic tree was constructed through MEGA11 [50].

### miRNA target analysis and crosstalk construction

this study also predicted the targets of miR-223 through software such as: miRDB (http://www.mirdb.org), TargetScanHuman 8.0 (https://www.targetscan.org/vert_80/), DIANA Tools (https://diana.imis.athena-innovation.gr/DianaTools/). To establish a genetic pathway, genetic crosstalk was constructed among the understudied genes, i.e., TPD52, KLF9, miR-223, and PKCε. KEGG genome database [51] was used, and further analysis of gene linkage was performed through STRING [52]. GeneMANIA [53] was used to analyze the intra-molecular interactions among the genes, while the molecular pathway was obtained via DAVID software [54].

## Results

### Prediction of tumor protein D52 (TPD52) tertiary structure

**Fig. 1:**
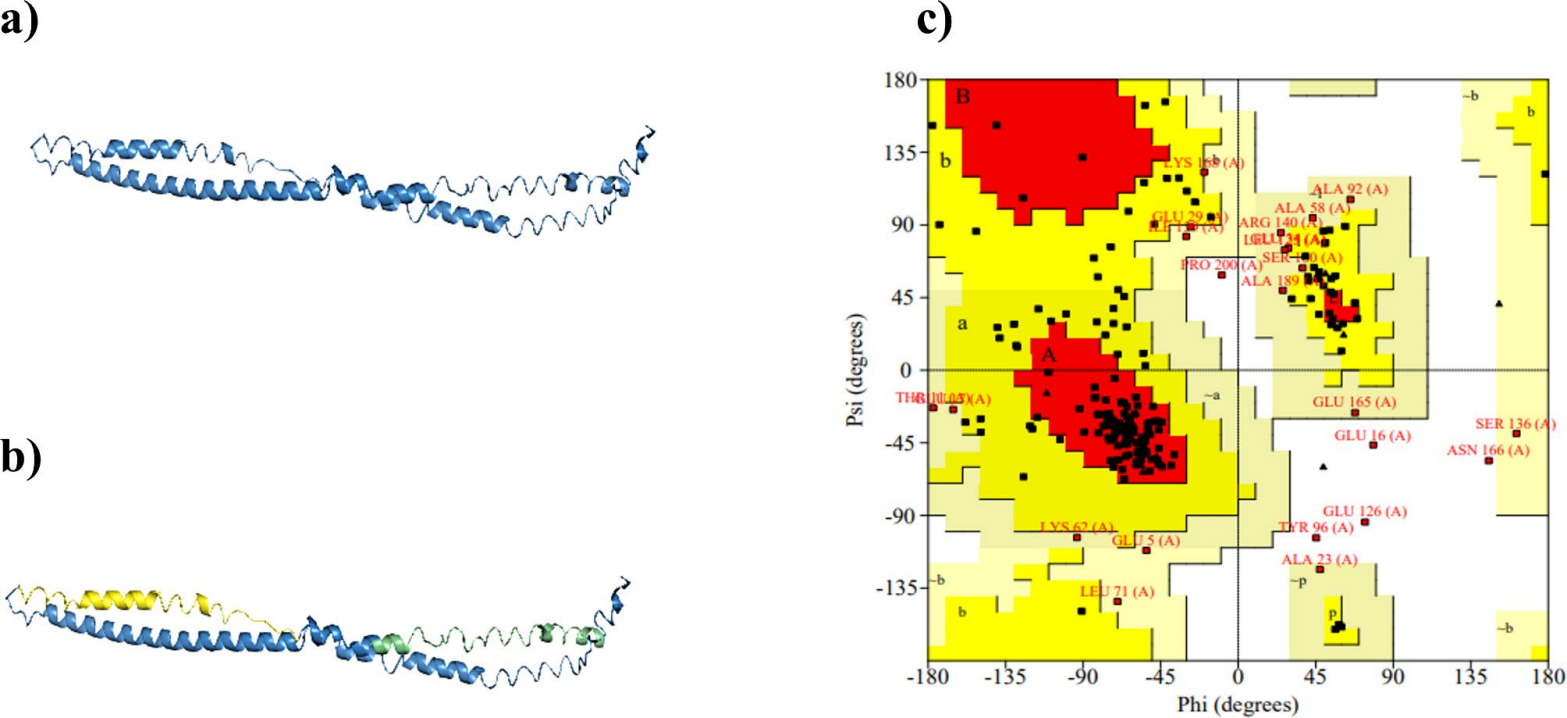
3-Dimentional structure of TPD52. **(a)** Model with highest C-score value.** (b)** Predicted motifs and regions of TPD52. Region highlighted in yellow represents the residues of coiled-coil motif, while the region colored with green represents disordered region. **(c)** Ramachandran plot analysis of TPD52 representing the quality of predicted structure

TPD52 is a 224 amino acid protein. The tertiary structure of TPD52 was predicted with I-TASSER. Five models were predicted with different Confidence-score values using *ab-initio* protein modeling, and the model with the highest C-score (-1.83) was selected for further analysis (Fig. 1a). Domain analysis from InterPro revealed that TPD52 contains a coiled-coil motif from amino acids 69–110 (Fig. 1b). This motif is composed of 2–7 α-helix, coiled together, forming a rope-like structure [55]. This protein motif is required for the interactions of TPD52 with other proteins. This domain is essential in the localization and tethering of TPD52 with cellular compartments and organelles like cytoplasm, Golgi bodies, and endoplasmic reticulum [56, 57]. TPD52 also contained a disordered region from residues 187–224 in the C-terminus (Fig. 1b). This region has an essential function regarding the rotation of proteins to acquire favorable 3-D orientations, enabling molecular interactions with other molecular partners. Both coiled motif and disordered regions are shown to have a role in the interactions of TPD52 with other interacting partners [58, 59].

To verify the quality of the predicted model, analysis was performed using the ERRAT tool [60], which showed that the quality of the predicted structure was high, with a score of 84.9. Ramachandran plots were also used to analyze the quality of predicted model (Fig. 1c). These plots were used for visualization of dihedral angles, i.e., phi (φ) and psi (ψ) angles of amino acids [61]. Ramachandran analysis for structural validation showed that 97.9% of the TPD52 residues were present in the allowed region of the plot while only 2.2% of the residues were in the disallowed region, verifying the quality of the predicted structure.

### Multiple sequence alignment

**Fig. 2:**
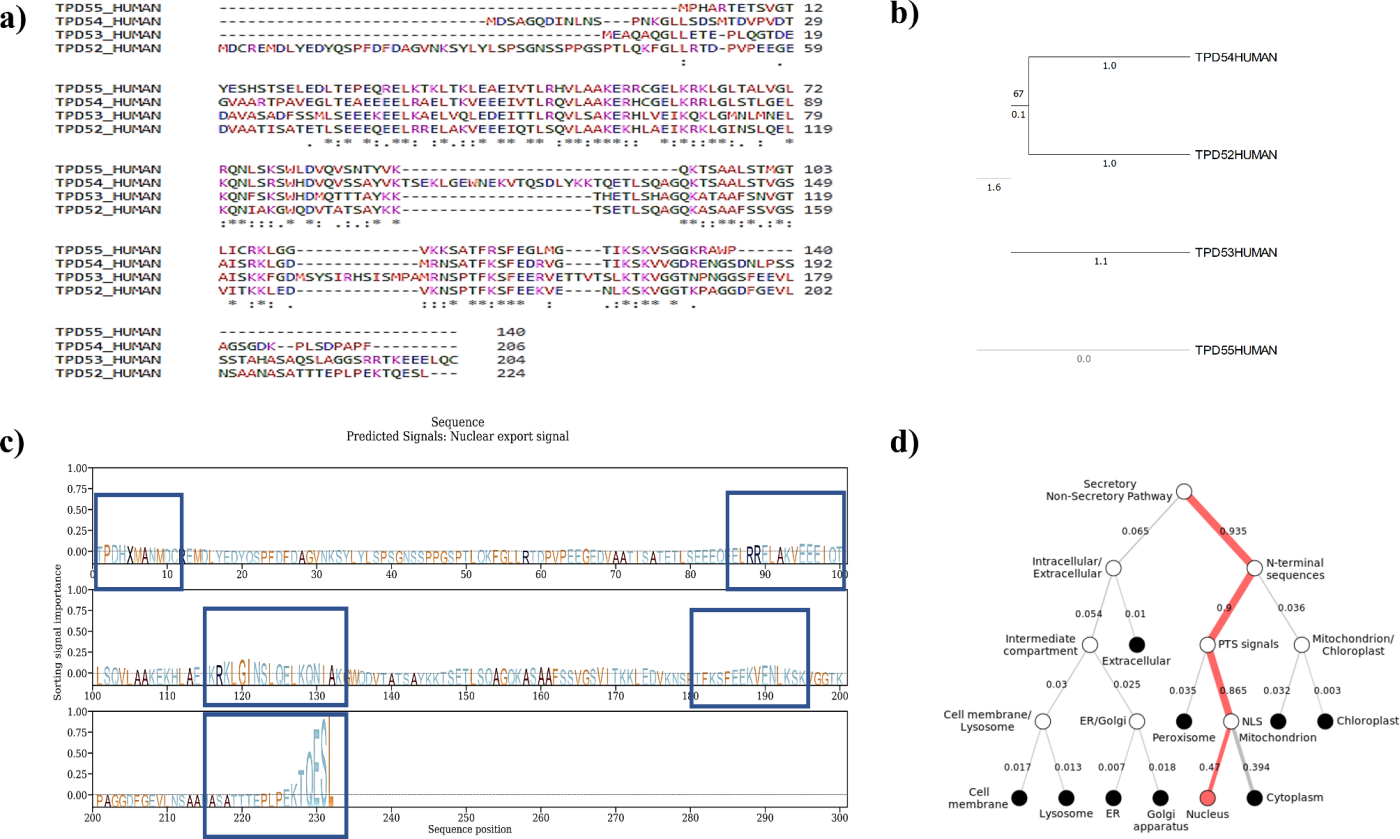
**(a)** Multiple sequence alignment of TPD family proteins depicting that certain sequences in the coiled coil motif and disordered region are conserved among all the proteins of this family. **(b)** Phylogenetic analysis of TPD family proteins. The evolutionary history was inferred using the unweighted pair group method with arithmetic mean (UPGMA). **(c)** Predicted signal displaying the sorting signal for TPD52. TPD52 contains nucleus localization signal and important regions for localization are highlighted. **(d)** Subcellular localization tree indicating that TPD52 primarily localize towards cytoplasm and nucleus

To analyze the evolutionary relationship among the residues of TPD family proteins, multiple sequence alignment (MSA) analysis was performed with Clustal Omega [62]. The results revealed that amino acid residues constituting the coiled-coil motif have the highest conservation among the TPD family proteins. Furthermore, specific residues in the disordered region were also shown to have evolutionarily conserved sequences, as depicted in (Fig. 2a).

### Phylogenetic tree construction

Phylogenetic analysis of the TPD52 was performed through MEGA11 [63] using the UPGMA phylogenetic tree to understand the evolutionary histories and conservation of different members of the TPD family (Fig. 2b). This analysis revealed that the TPD family members are organized into groups based on their evolutionary sequence conservation and structural characteristics. The first group includes TPD52 and TPD54. These proteins are associated with various cellular processes, such as exocytotic excretion and vesicle trafficking [64]. The second clad contains only a branch representing TPD53, which is involved in multiple cell cycle regulation pathways. Lastly, the third clad includes TPD55. However, its role in regulating cellular functions and pathways still needs to be determined.

### Subcellular localization

Intracellular translocation analysis of TPD52 revealed that TPD52 is predominantly present in the cytoplasm, with a Probability score of 0.6. TPD52 was also identified to harbor nuclear localization signals (Fig. 2c and d). Moreover, results suggested localization of TPD52 to several vesicular organelles, including ER, Golgi bodies, mitochondria, lysosome, and cellular membranes. Results are summarized in Table 3.

**Table 3:** Subcellular localization of TPD52

Localization	Probability
**Cytoplasm**	0.3164
**Nucleus**	0.2924
**Extracellular**	0.0065
**Cellular membrane**	0.016
**Mitochondria**	0.0622
**Plastid**	0.0145
**Endoplasmic reticulum**	0.033
**Lysosome**	0.0266
**Golgi body**	0.1788
**Peroxisome**	0.0536

### Pathway establishing a crosstalk between TPD52, KLF 9, PKCε, and miR- 223

According to the results of KEGG and String analyses, TPD52 and KLF 9, PKCε, and miR- 223 are interconnected and have a role in the AKT pathway. The DAVID-obtained route demonstrated that PKCε regulates Ras/Raf signaling by acting upstream of it. Additionally, PKCε has been shown to play a role in cardiomyocyte remodeling via activating the receptor tyrosine kinase (RTK) linked Ras/Raf pathway [37].

**Fig. 3:**
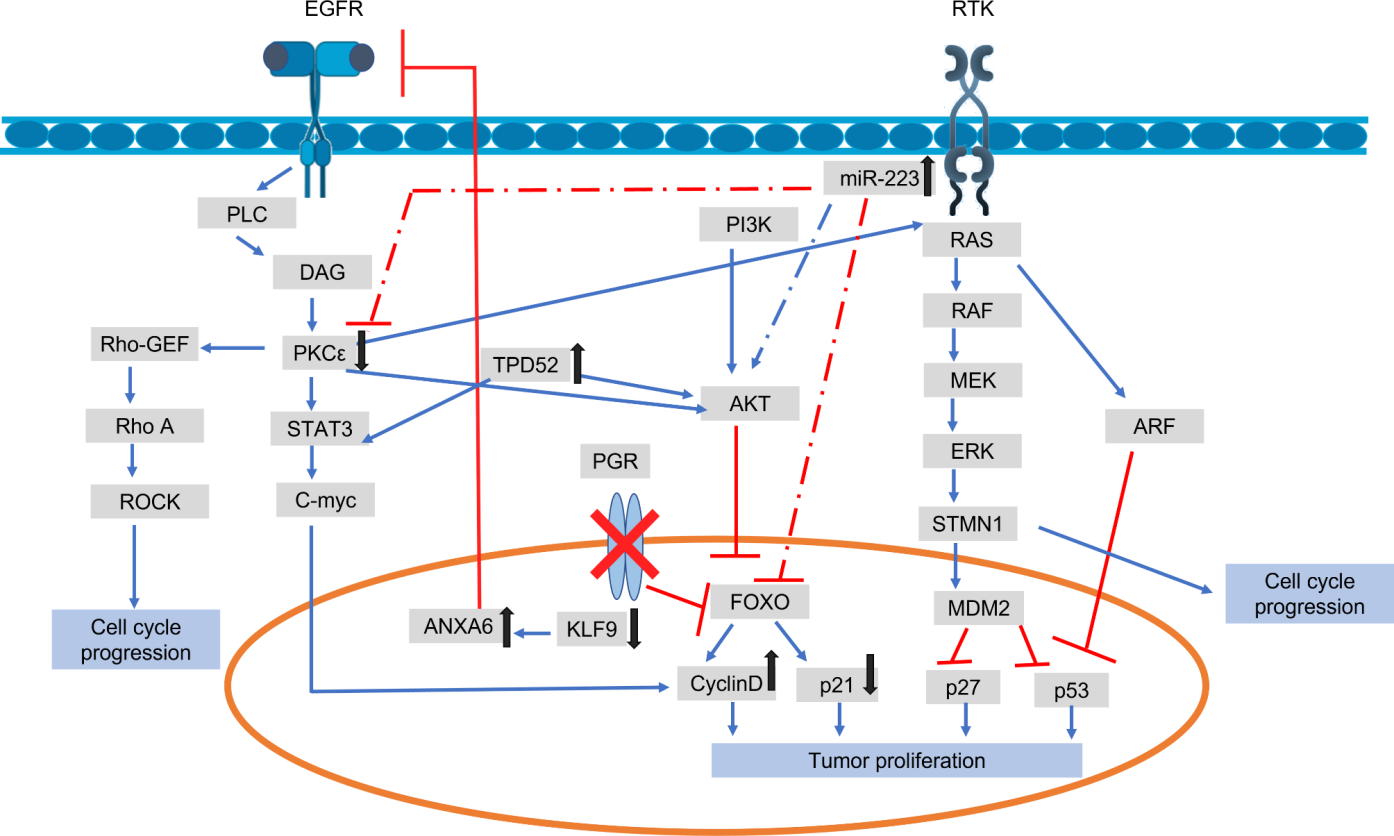
Pathway showing crosstalk between TPD52, KLF9, PKCε & miRNA 223

Nucleus-localized PKCε has been shown to have a regulatory function with the signal transducer and activator of the transcription 3 (STAT3) gene, which activates the c-myc gene, leading to stimulation of cyclin D activity and increased cell cycle progression [65, 66] (Fig. 3). PKCε regulatory role in prostate adenocarcinoma has also been established [67, 68]. Moreover, a recent study also determined the role of TPD52 in activating STAT3 [18]. Hence, the transcriptional activity of STAT3 is regulated by PKCε, TPD52, and Rho-kinases. PKCε also promotes breast cancer metastasis by inducing Rho GTPases activation, which possesses putative phosphorylation sites for PKCε and is its potential effector [69]. Pathway analysis indicated that RhoA GTPases are found downstream of PKCε. Our finding is supported by evidence that ERK phosphorylation in the Ras/Raf pathway is caused by the induction of Rho GTPases, a downstream target of PKC [39]. Our findings demonstrate that PKC functions in the AKT pathway since it is positioned upstream of TPD52 and can activate the route, perhaps increasing Tumor development and dissemination. PKC’s role has been proven by its ability to phosphorylate AKT at the Ser473 location [70]. By regulating proteins such as cyclin D1, p21, p53, and p27, the AKT pathway is known to regulate cell development and progression [71]. Through the activities of TPD52 and PKC, the PI3K/AKT signaling pathway promotes cell growth and proliferation by suppressing the transcription factor FoxO, an AKT downstream target. FoxO is rendered inactive by phosphorylation, resulting in the overexpression of cyclin D1 and the downregulation of the cell cycle inhibitor p27. Consequently, the PI3K/AKT pathway downregulates cell cycle regulators such as CDKI and p27 [72].

This work demonstrates that reduced expression of KLF9 has a detrimental effect on the growth-promoting hormone PGR, inhibiting the transcription factor FOXO and promoting Tumor development. Another research [73] verifies this by revealing that the absence of KLF9 inhibits PGR and FOXO signaling in endometrial cells, boosting oncogenesis and Tumor spread. In addition, decreasing levels of KLF9 increase the expression of ANXA6, which leads to the downregulation of EGFR and a reduction in PKC activation [74]. Our pathway also indicates that higher miR-223 expression activates STMN1 and inhibits FOXO. In gastric cancer, overexpression of miR-223 is associated with decreased FOXO expression and suppression of cyclin D, p21, and p27 [75]. In addition, it is known that miR-223 promotes the PI3K signaling pathway, which generates PIP3 in the cell membrane and activates the AKT pathway. In addition, it has been shown that miR-223 interacts with PKC, leading to its inactivation. Recent research has emphasized the significance of overexpressed miR-223 in activating AKT and increasing ovarian cancer Tumor development [76].

### Expression of TPD52, KLF 9, PKCε and miR-223 in blood of ovarian cancer patients

**Fig. 4:**
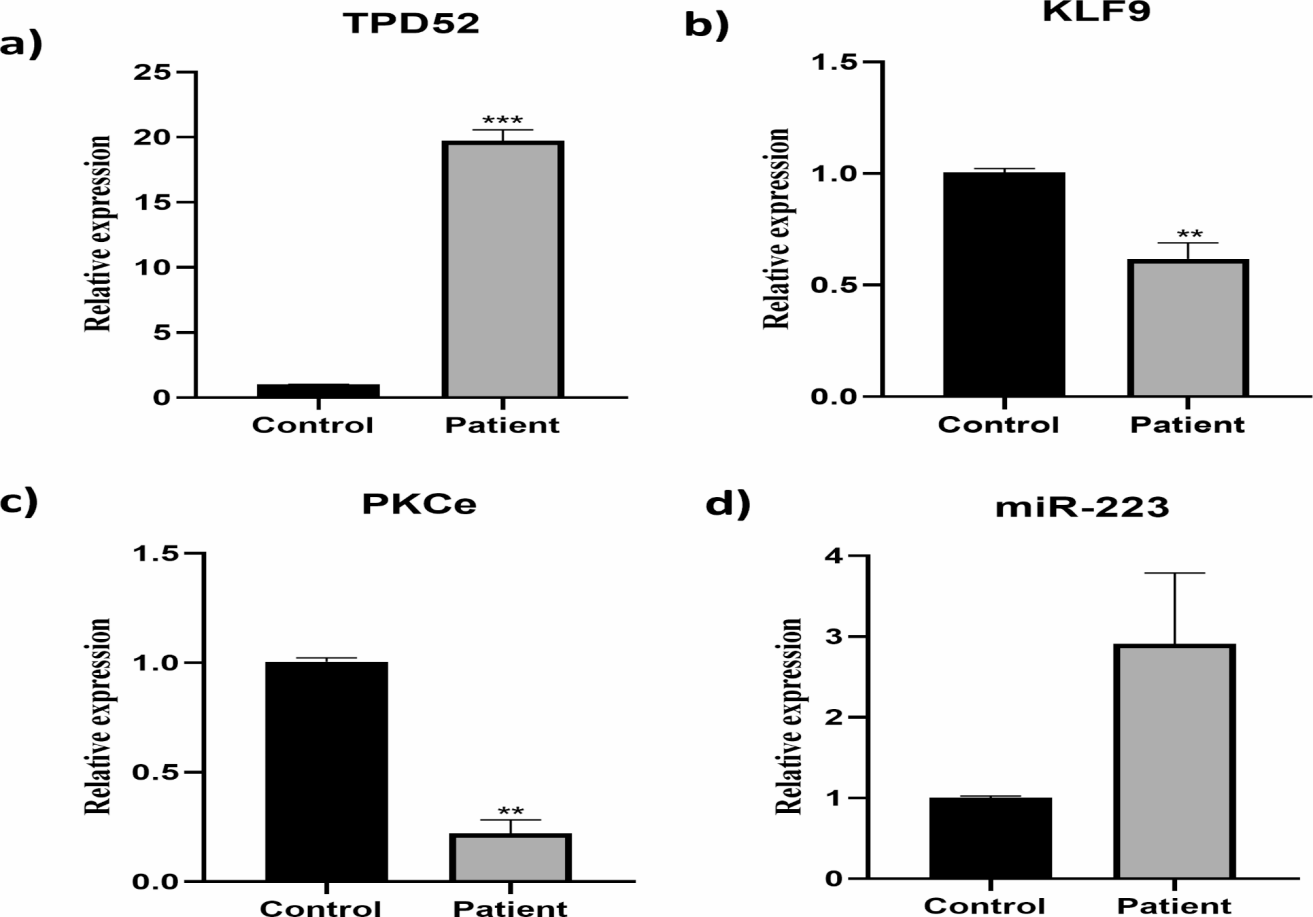
Expression of TPD52, KLF 9, miR- 223 and PKCε in blood of ovarian cancer patients. TPD52 expression is up-regulated (***p = 0.0002) (A); KLF 9 expression is fold decreased (***p = 0.0006) (B); miR-223 expression is increased (***P = 0.0003) (C); PKCε is down-regulated (**P = 0.007) (D) in ovarian cancer patients. Illustrative data were presented as mean ± SEM of triplicate experimentations. Statistical significance was measured by ordinary 2-way ANOVA.

Our pathway analysis reveals that TPD52 influences the activation of many signaling proteins, hence increasing ovarian cancer. To address this, we compared the expression of TPD52 to KLF 9, PKCε, and miR-223 in ovarian cancer patients and healthy persons. Using Realtime PCR, we evaluated the expression of these four genes in 150 patients and 150 healthy controls. Compared to healthy controls, the expression of TPD52 (19.79 ± 0.42) was higher in ovarian cancer patients. Moreover, KLF9 expression was considerably lower (0.61 ± 1.6) in ovarian cancer patients than in healthy controls, but the miR-223 expression was up-regulated (2.9 ± 4.0), and PKCε expression was reduced in ovarian cancer patients. Overall, our data indicate that TPD52 and miR-223 are greater in ovarian cancer patients than in healthy persons, but the expression of KLF9 and PKCε is lower (0.2 ± 5.7) in ovarian cancer patients than in healthy individuals **(**Fig. 4**)**.

### Correlation of TPD52, KLF9, PKCε and miR-223 expressions with clinical features in ovarian cancer

**Fig. 5:**
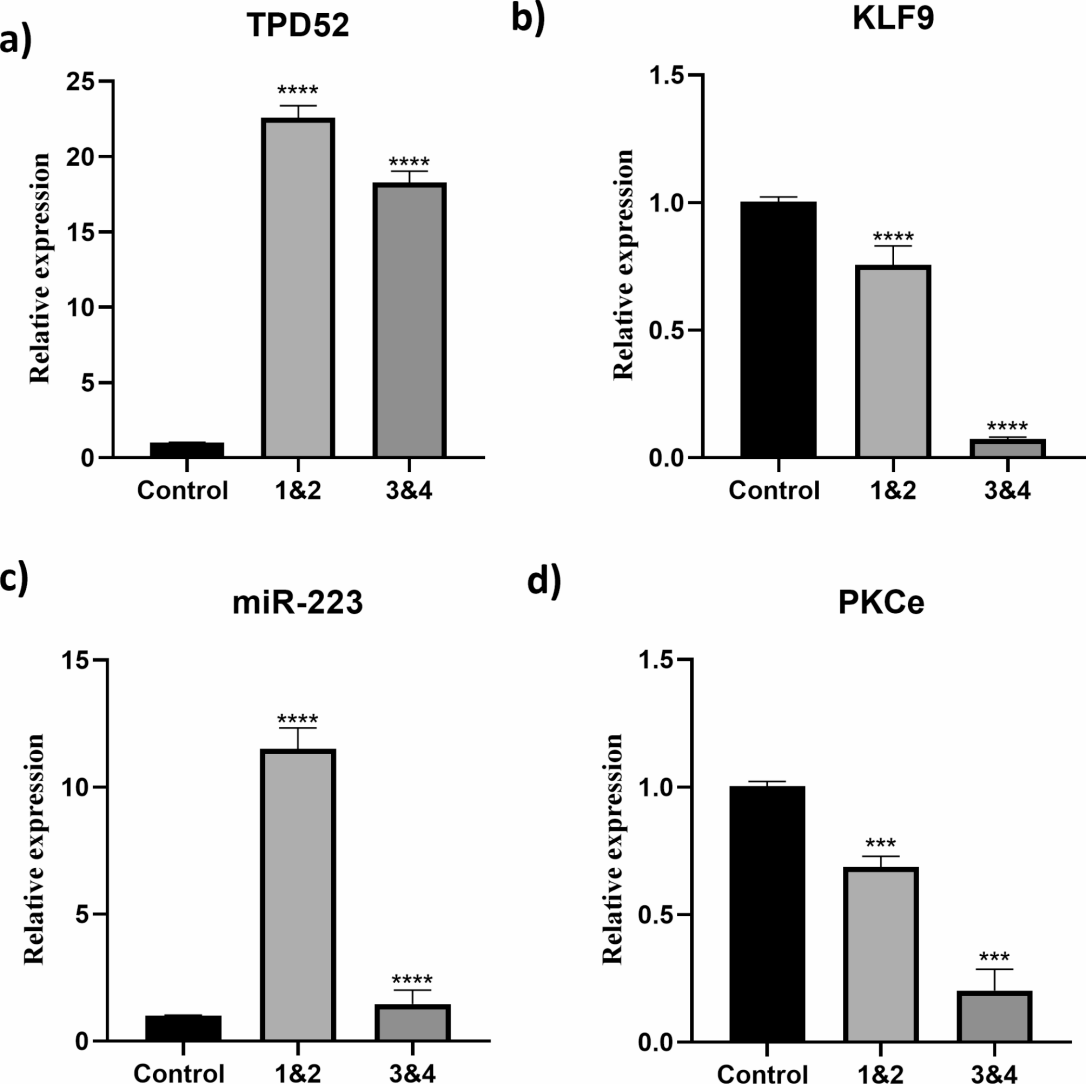
Correlation of gene expression with clinical stage of ovarian cancer. Correlation of TPD52 expression with **(A)** tumor stage in ovarian cancer patients. Correlation of KLF 9 expression with **(B)** tumor stage in ovarian cancer patients. Correlation of miR-223 overexpression with **(C)** tumor stage in ovarian cancer patients. Correlation of PKCε overexpression with **(D)** tumor stage in ovarian cancer patients. Illustrative data were presented as mean ± SEM of triplicate experimentations. Statistical significance was measured by ordinary one-way ANOVA (****p < 0.0001)

**Fig. 6:**
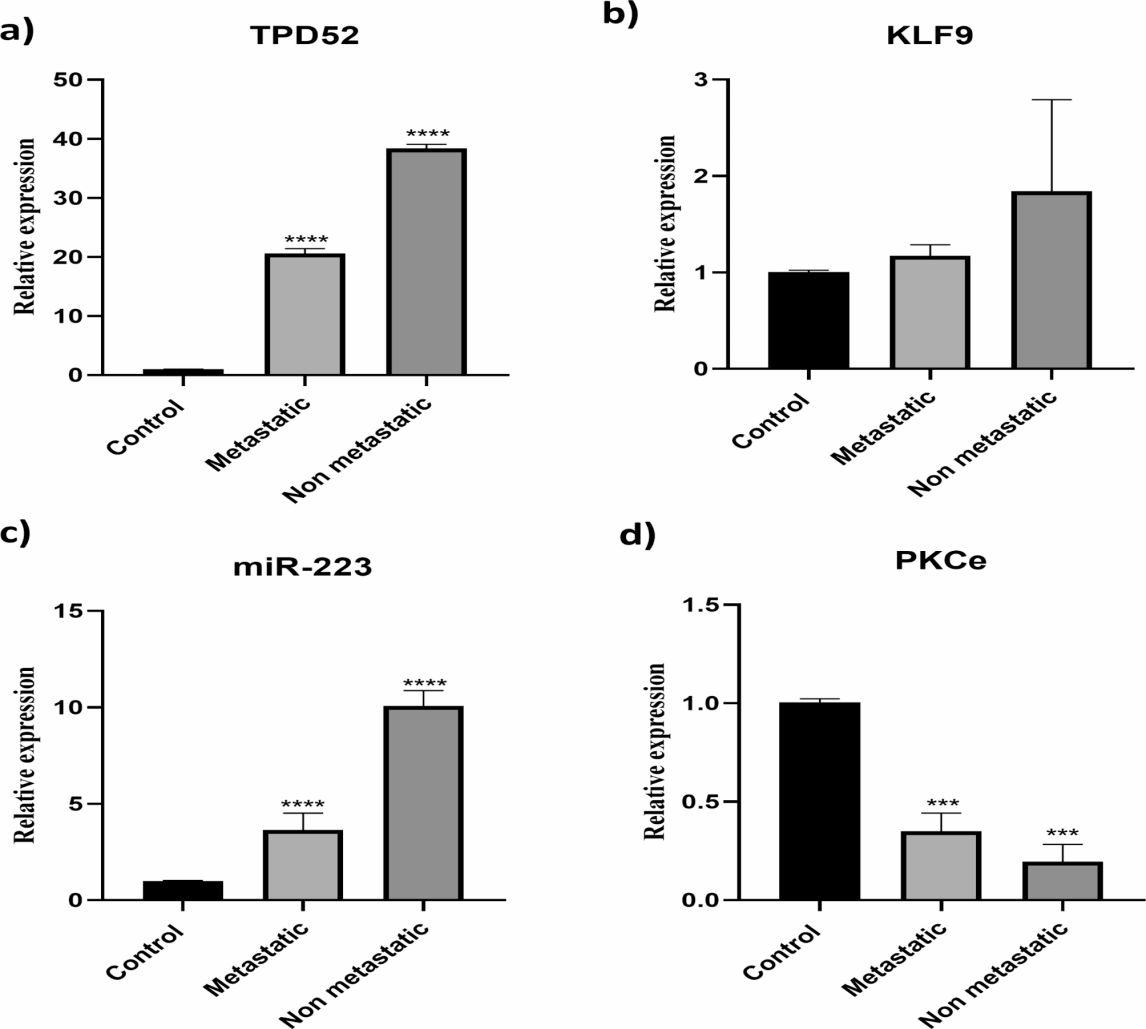
Correlation of gene expression with metastasis state of ovarian cancer. Correlation of TPD52 expression with **(A)** metastasis in ovarian cancer patients. Correlation of KLF 9 **(B)** metastasis in ovarian cancer patients. Correlation of miR-223 **(C)** metastasis in ovarian cancer patients. Correlation of PKCε **(D)** metastasis in ovarian cancer patients. Illustrative data were presented as mean ± SEM of triplicate experimentations. Statistical significance was measured by ordinary one-way ANOVA (****p < 0.0001)

TPD52, KLF9, PKCε, and miR-223 expression levels were compared between ovarian cancer patients and healthy controls. We also looked at how these gene expression levels correlated with different clinical characteristics of ovarian cancer patients. We examined the fold changes and expression levels of these genes in connection to several clinical features, including treatment status, metastatic and non-metastatic groups, early-stage (I-II) and advanced-stage (III-IV) groups, and early-stage (I-II) and advanced-stage (III-IV) groups (chemotherapy, radiotherapy, or chemoradiotherapy). Figures 5 and 6 depict the analyses’ findings, which were shown to be statistically significant (P 0.001) across all patient groups.

TPD52 and miR-223 expression were considerably greater in early-stage Tumors and non-metastatic patients compared to advanced-stage Tumors and distant metastatic patients, according to the research. In contrast, the expression of KLF9 was dramatically decreased in advanced-stage Tumors and distant metastases, although it was marginally enhanced in early-stage Tumors and non-metastases. Similarly, the expression of PKCε was much lower in early-stage Tumors and non-metastatic patients than in advanced-stage Tumors and distant metastatic patients. TPD52 expression was specifically 20.6-fold higher in distant metastases and 18.53-fold higher in advanced-stage malignancies. 0.06-fold decreased KLF9 expression in advanced-stage Tumors, but 3.6-fold elevated the miR-223 expression in distant metastases. 0.2-fold lowered PKCε expression in the distant metastasis group and 0.11-fold in advanced stage Tumors compared to the non-metastatic and lower-stage Tumor groups, in which PKCε expression was reduced by 0.1-fold and 0.6-fold, respectively. These outcomes are shown in Figs. 5 and 6.

### Specificity of targeted genes and miR-223 for diagnosis of ovarian cancer

**Fig. 7:**
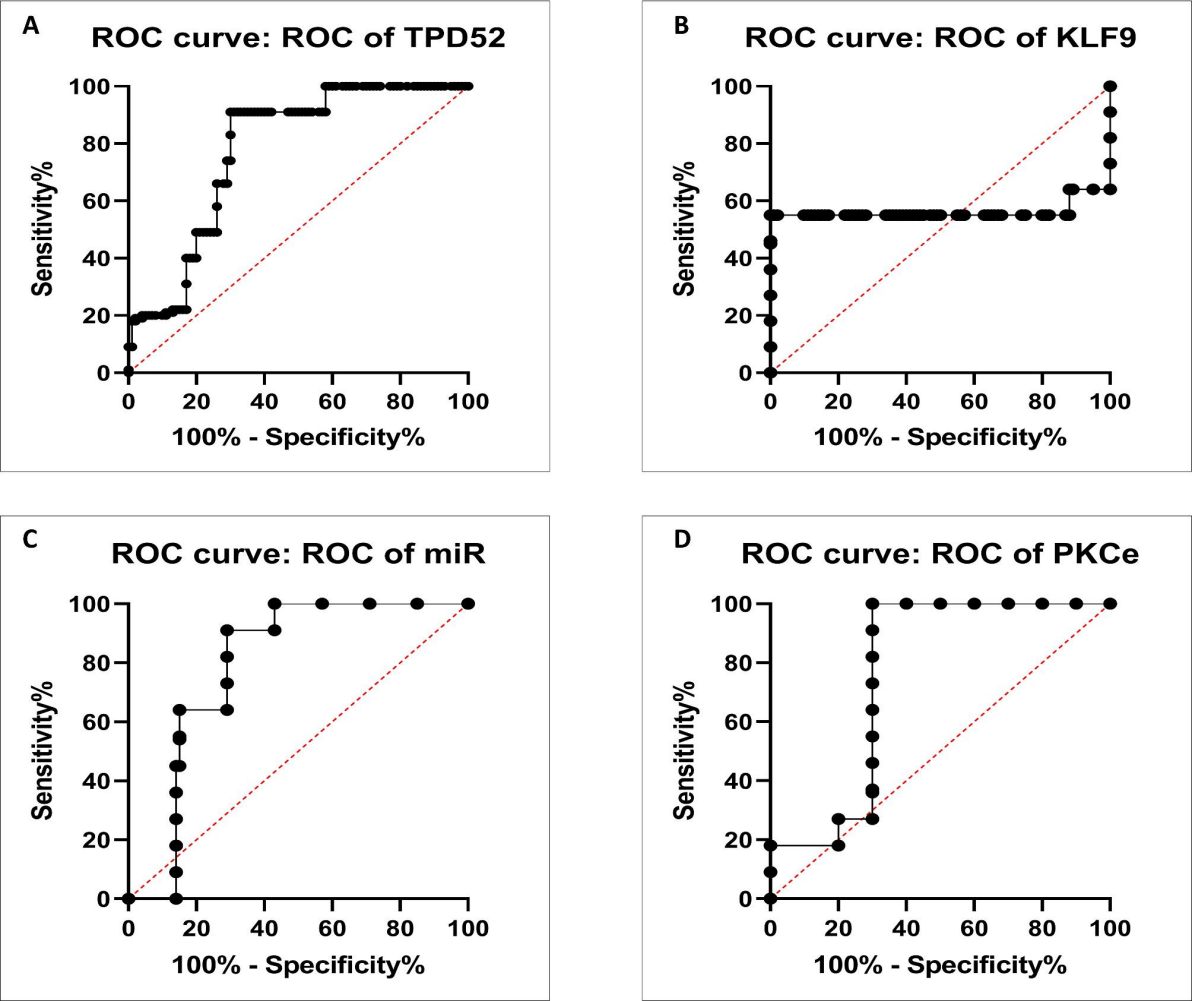
Specificity of miR-223 and target genes TPD52, KLF9, and PKCε expression in ovarian cancer diagnosis. Receiver operating characteristic (ROC) curve analysis for miR-223, TPD52, KLF9, and PKCe predicted elevated risk of ovarian cancer cancer. **(A)** TPD52 AUC = 0.7769; 95% CI = 0.7100– 0.8438. **(B) **KLF9 AUC = 0.5608; 95% CI = 0.4652–0.6564. **(C)** miR-223 AUC = 0.7915; 95% CI = 0.7213– 0.8617. **(D)** PKCε AUC = 0.7630; 95% CI = 0.6883–0.8377.

To verify the association between the detected blood-based biomarkers including miR-223, tpd52, KLF9, and PKCϵ with ovarian cancer, ROC analysis was carried out and ROC curves were created as shown in Fig. 7. This study also calculated the area under the ROC curve (AUC) as well as the 95% confidence intervals (CI) were established.

## Discussion

The genital tract malignancies are found to be the third most deadliest cancer among females. Ovarian cancer ranks as the seventh most frequent cancer incidence and the fifth leading cause of cancer-related mortalities among the women, globally [77]. Ovarian cancer is referred to as “the silent killer” due to the unavailability of early diagnostic and screening techniques—the majority of females diagnosed with ovarian cancer present signs and symptoms. Only a minor percentage of females diagnosed with the lower-stage disease will be asymptomatic. Because of the common and non-specific nature of the tumorous ovaries, difficulties are faced in establishing a diagnosis. Most females with tumorous ovaries are diagnosed only after the cancer has metastasized, which underlies the high mortality rate. According to an estimate, almost 70–75% of women with tumorous ovaries are diagnosed at an advanced stage [78].

Despite the emergence of numerous screening approaches for the diagnosis of the ovarian cancer. The overall rate of mortality remains high (87%). Diagnosis of ovarian cancer involves different tests. For instance, CA-125 is used as a biomarker for ovarian carcinoma. But its levels in serum may also be linked with endometriosis, breast cancer, lung cancer, pancreatic cancer, colon cancer, pelvic inflammatory disease, pregnancy, and menstruation [9]. Hence, discovering the molecular mechanisms underlying cancer development and improving screening and diagnostic strategies for early diagnosis of ovarian cancer are essential goals in ovarian cancer research.

The current study objective was to determine the differential expression of TPD52 along with PKCε, KLF9, and miR-223. Previous investigations studies the role of these genes in different malignancies and found their association with cancer proliferation, invasiveness, and treatment resistance. However, the differential expression of these genes has never been studied before. Therefore, in present investigation, we determined the co-expression of TPD52, PKCε, KLF9, and miR-223 in ovarian cancer. That furthered our understanding of cancer mechanism.

TPD52 mRNA levels were observed to be upregulated in ovarian cancer patients relative to control. This is consistent with earlier findings demonstrating elevated expression of TPD52 in several cancer types, including breast, pancreatic, multiple myeloma, prostate, Burkitt’s lymphoma, and melanoma [14]. However, TPD52 expression is also determined to be downregulated in few cancers, including lung, papillary renal cell, and liposarcoma. This uneven expression pattern across various cancer types has led to the designation of TPD52 as a “controversial gene” [15].

In KLF9, expression levels in ovarian cancer were much lower than in healthy controls. The expression down-regulation of KLF9 has been demonstrated in numerous cancers including endometrial cancer, colorectal tumors, breast cancer, and hepatocellular carcinoma [28, 79]. Similarly, the relative proteins of KLF9 also have been reported to have expression dysregulation in various malignancies. Reduced KLF4 expression is related to poor survival in gastric cancer [80]. KLF6 expression is elevated in ovarian malignancies, KLF8 promotes cellular proliferation in HCC, gastric, and glioma carcinomas, while KLF3 and KLF14 have been reported to inhibit tumor growth in breast and brain tumors, respectively [42, 43, 81].

Circulating microRNAs, abundant in the blood, have been associated with the development and prognosis of many forms of cancer. Studies have shown that assessing the expression levels of these miRNAs may be helpful in cancer diagnosis and therapy [82]. MiR-223 has been identified as being overexpressed in ovarian and gastric cancer patients. This overexpression has been linked to elevated proliferation of cells and reduced cell death in gastric cancer [83]. In contrast, the decreased miR-223 expression has been associated to cancer subtypes, including esophageal, leukemia, gastric, and colorectal cancer [84].

The data from this investigation revealed that MiR-223 targets and alter the expression of its target genes which are PKCε and KLF9, as can be seen by their decreased expression values. Furthermore, KLF9, which acts as a transcriptional repressor, is now unable to repress the transcription of its target gene i.e. TPD52 and its expression levels are elevated. A similar study has also reported that elevated level of miR-223 alleviates the expression levels of PKCε and KLF9, and can serve as potential biomarker for cervical cancer [85].

PKC, a protein kinase C family member, is well-studied and well-known for its involvement in carcinogenesis [34]. Despite having high quantities of this gene in their blood, PKCε expression was downregulated in ovarian cancer patients in comparison to healthy individuals, according to our study. However, PKC is often upregulated in the breast, lung, and prostate [86]. In addition, research indicates that PKC is involved in tumor metastasis [39, 43]. Furthermore, its genetic variations are also depicted to have the potential to be a possible cause of its pathogenicity [59, 87].

TPD52 expression was greater advanced-stage ovarian cancer and those with distant metastases compared to healthy controls. Intriguingly, TPD52 levels were significantly greater in individuals with less advanced tumors and no evidence of metastasis. This shows that TPD52 expression is more significant in early-stage and non-metastatic ovarian cancer than in later-stage cancer and distant metastases. Given that TPD52 expression is elevated in every occurrence of ovarian cancer, it may be a valuable biomarker for early disease detection. Expression of KLF9 was lower in advanced-stage cancers, those with distant metastasis, and lower-stage tumors without metastasis compared to controls. In esophageal squamous cell carcinoma, lower KLF9 expression was associated with cancer metastasis and tumor stage/size [88].

The current investigation has evaluated the expression of genes in the blood sample from ovarian cancer patients. As miR-223 is an oncogenic circulating miRNA and its expression level was found to be elevated in this study. Previously, several studies have reported that the elevated plasma levels of this particular miRNA are associated with various kinds of cancers. Since the other genes whose expression has also been determined are the direct target of miR-223, therefore, their expression has also been modulated. Although this expression profiling was conducted on the blood samples from ovarian cancer patients, the expression pattern of these genes can give a clear picture of cancer pathogenesis.

The levels of miR-223 were observed to be elevated in advanced-stage cancers (1.8 ± 3.6), tumors with distant metastasis (1.31 ± 2.0), lower-stage tumors without metastasis (2.45 ± 3.3), and non-metastatic tumors (2.1 ± 3.6). This shows that the overexpression of miR-223 may be a valuable biomarker for diagnosing ovarian cancer. In addition, all of the genes investigated in this study are engaged in many cancer-associated signaling cascades, including the PI3K/AKT pathway, nuclear factor-B signaling, WNT–catenin pathway, and Ras signaling [89], which makes them prospective diagnostic biomarkers and anti-cancer therapeutic targets. The evaluation of these biomarkers’ diagnostic specificity was further investigated by the ROC curve analysis. However, additional analysis on a bigger sample size and at the level of proteins is required to determine their clinical importance.

## Conclusion

The expression of genes TPD52, PKCε, KLF9, and miR-223 in the peripheral blood of ovarian cancer patients was studied in the present investigation, that indicated the expression of TPD52 as well as miR-223 were elevated. In contrast, the levels of KLF9 and PKC were decreased. This led us to conclude that TPD52 and miR-223 are likely involved in the development of ovarian cancer. These shifts in the expression of genes have been connected to the development of cancers. As a consequence of this, these genes have the potential to be used as biomarkers to diagnose and assess ovarian cancer. Additional studies on the roles, pathways, and interactions of TPD52, KLF9, miR-223, and PKC may give valuable insights into the progression of ovarian cancer and assist in identifying novel therapeutic targets. This understanding could be helpful in the development of treatments that are more successful for ovarian cancer.

## Data Availability

All the relevant data has been provided in the manuscript are available from the corresponding author on reasonable request.
